# Literacy, power, and affective (dis)encounter: An ethnographic study on a low-income community in Spain

**DOI:** 10.1371/journal.pone.0252782

**Published:** 2021-06-04

**Authors:** Elena Guichot-Muñoz, María Jesús Balbás-Ortega, Eduardo García-Jiménez

**Affiliations:** 1 Department of Language and Literature Teaching, University of Seville, Seville, Spain; 2 Department of Didactics and Educational Organization, University of Seville, Seville, Spain; 3 Department of Education and Research, University of Seville, Seville, Spain; University of Granada: Universidad de Granada, SPAIN

## Abstract

In areas of social exclusion, there are greater risks of facing discrimination at school. The teaching-learning processes may contribute toward the perpetuation of this inequality. This research analyzes a literacy event that takes place in a low-income school in Southern Spain. The new literacy studies have come to examine how power relationships and affective bonds work in such literacy practices. An ethnographic method was followed to facilitate a deeper understanding of multimodal literacy. Further, a social semiotics multimodal approach was adopted to analyze the meaning-making social process that takes place in the classroom. The participants comprised two teachers and 17 children, whose ages range from 5 to 7 years. Data were collected in the form of reports, audio recordings, video recordings, and photographs over a two-years period. The results obtained have revealed that the children have been taught writing and reading through a dominant orthodox model that fails to consider the community’s and families’ cultural capitals. They also show that the literacy process does not grant any affective quality. Neither is there an authentic dialogic space created between the school and the community. This lack of dialogue generates an inequality in the actual acquisition of comprehensive reading and writing skills at school, with instances of groups exclusion, owing to the anti-hegemonic practices of knowledge acquisition.

## Introduction

In disadvantaged environments where cultural capital and “knowledge funds” [1] are disregarded in schools, a systematic practice of “colonial difference” is established [2]. It is an educational domination strategy that has its origination in imperialism [3], wherein the clashes with the global and democratic principles are supposedly born in the knowledge era. Multiple studies have advocated for the recognition of other modes of literacy related to diverse cultures in schools [4–8] with a view to eradicate the discriminatory practices that are inherent in colonial systems. The new literacy studies [9] are born as a part of the initiatives intended toward studying literacy from a social, institutional, and historical point of view which could elucidate on the power mechanisms that are quite often disguised as educational innovation in the 21^st^ century. This novel approach considers the need for emphasizing on the material and non-material -i.e. (im)material- aspect of literacy: how the texts lead us to a relationship between the texts and the felt experience of the participants that goes beyond the material [10]. The new research endeavors that arise from this movement analyze literacy as a social practice [11]. Moreover, they conceive this process as a practice that hosts day-to-day creativity [12]. From the “paradigmatic” standpoint [13], this approach focuses on the literacy practices in certain social, cultural, and economic contexts [9, 14]. The approach under study goes further than the psycholinguistic or structuralist analyses that have prevailed in the field of communication skills. Not only does this approach completely revolutionize the concept of literacy [15], but it also associates literacy with an increased involvement and collaboration and a greater democratic spirit in the way literacy is used, shared, and taught [11].

From this standpoint, our work delves into the literacy event as an “affective encounter” with the text, the individuals, the space, and everything that goes on at a given moment. Our work refrains from considering this to be an isolated activity that refers solely to the cognitive aspects. In line with the work carried out by Leander & Ehret [16], this study analyzes the humanizing and dehumanizing influences that literacy exerts on schools and examines the affective aspects of the literacy teaching role at the individual and community levels. The purpose of such kind of research is to find the movements, rhythms, atmospheres of social life that go beyond the theoretical scope offered by constructivism alone [16]. The Affect Theory’s most interesting potential, when it comes to literacy, is the development of sociocultural perspectives that examine the textures and rhythms of the literacy teaching-learning process through an in-depth exploration of the interactions and structures that make up the process [17]. Likewise, it studies the social value attributed to the cultural written practices by different event participants [18] and investigates how the participants and the literacy event relate to each other from a theoretical post-colonial perspective [2, 19].

Classroom teaching-learning processes are understood as the material (cognitive and affective) expression that exists between teachers and students, as well as amongst the array of resources available at a given time in each situation. This stems from the Social Semiotics’ Multimodality theory [20]. It provides a view of multimodality that spans the analysis of a broad range of resources, other than verbal discourse, as in a school classroom setting. It is for this reason that the case study presented here analyzes how teachers and students, turn not only to writing, but also to gesture, image, stance, speech, media, and other modes to develop reading and writing habits. In accordance with Halliday [21] and Kress [22] theories, the analysis resorts to meaning networks that are “traces of a sign-maker’s decision-making about what is the most apt and plausible signifier for the expression of the intended meaning in a given context” [23, p44].

For the sake of supplying a context for this specific experience, it should be noted that the radio programs broadcasted from school are intended to promote student literacy. Notwithstanding this, the experience addressed by our study ended up thwarting the affective encounter between families and the school. The weekend before the date the program was scheduled to be broadcasted, the negative affective encounter reached the breaking point when the father of a female student (J.) tore up the radio script after noticing that his daughter was distressed and crying because of her inability to read the text. The question that arises is “Why did the radio program fail to attain its objective and end up causing a conflict between the family and the school?”

Given this theoretical framework and context, we formulated the following research questions:

RQ1: How is power exerted in classrooms to impose a dominant literacy model?
RQ 2: How is this model designed and developed through the literacy events that take place in the primary education of a community, which is at the risk of social exclusion?
RQ 3: With the literacy event being broadcasted on a radio program, what form does the affective encounter between the text, the students, and the spaces (school, home) take?

## Study design

This study was carried out by following an ethnographic methodology. More specifically, it is developed according to an ethnographic perspective [24], because it is focused on the study of particular aspects of every life and cultural practices of a social group guided by theories of culture and research practices from anthropology and sociology [25]. The literacy events that revolve around the broadcasting of a radio program by the students are those specific aspects of every day life, and the theory of affect is the socio-cultural theory that guides our study [16].

The research design used is a single-case study, a type 1 or holistic design in the terminology used by Yin [24]. This design is holistic because it focuses on one case and context–one classroom-, and a unit of analysis–a radio program-. This single-case study is an appropriate design when a critical case to the theoretical propositions of the affect theory is selected. Regarding the threats to validity, “a single-case study is analogous to a single experiment, and many of the same conditions that justify a single experiment also can justify a single-case study” [24, p51].

### Participants

A critical single-case was selected to determine whether the affect theory propositions are correct or whether some alternative set of explanations might be more relevant. The selection of critical cases is a kind of purposive sampling “aims at those cases which are particularly important for the functioning of the program to be evaluated” [25, p122].

The participants were two teachers, an experimented schoolteacher in charge of the classroom and a retired teacher who rules the radio program, and 17 children (ages between 5 and 7 years) from an urban elementary school located in a city in southern Spain. The urban area is clearly exceptional as it represents the most deprived neighbourhood in Spain in terms of per capita income according to the recent study of urban indicators (Urban Audit) by the National Institute of Statistics (INE). Due to the precarious situation of this zone, by ministerial order, an Integral Plan was elaborated in 2006 where a socio-educational diagnosis was carried out, indicating the following characteristics:

- Conflictive environment, problems in the neighborhood coexistence, lack of social skills, culture of impunity, insecurity
- Lack of awareness and social participation.
- Family situations of social risk.
- High rate of unemployment, precarious and low qualification employment, underground economy.
- Low academic level.
- High rate of population without academic or professional qualification.—Significant rate of illiteracy.
- Low involvement of parents.
- Abandonment of the neighborhood by the authorities.
- Barriers in the urbanistic design of the neighborhood, especially for the closing of the educational facilities. This, in addition, comes conditioned by the lack of response and solution to the problems of security of neighborhood [26, p86].

The situation has not evolved to any great extent according to the latest diagnosis of Areas in Need of Social Transformation (2019–2023) [27]. In terms of the training received, the majority of the students’ parents did not complete their primary education. Their homes have no books, pencils, or notebooks; the written texts have little relevance in their daily lives.

Although a majority of the students enrolled in the school are not of roman ethnicity (only 20%), Roma culture is predominant among the students in the classroom involved in this research. In this case, the concept of roman culture is understood as it is included in the Report of the Special Rapporteur on minority issues:

The term “Roma” refers to heterogeneous groups, the members of which live in various countries under different social, economic, cultural and other conditions. The term Roma thus does not denote a specific group but rather refers to the multifaceted Roma universe, which is comprised of groups and subgroups that overlap but are united by common historical roots, linguistic communalities and a shared experience of discrimination in relation to majority groups. “Roma” is therefore a multidimensional term that corresponds to the multiple and fluid nature of Roma identity [28, p3].

In the class that is the object of our study children have a high degree of absenteeism, with many of them not attending school one or two days a week. Most of them do not feel the need to go to school and exercise remarkable opposition against the school. Some families state that they take their children to school to ensure that they do not lose their children’s parental rights or ensure that their children benefit from services such as the school canteen.

At the literacy level, some children are still unable to distinguish vowels, they confuse ‘e’ with ‘i’ or ‘t.’ Other children either do not segment syllables in words or find it hard to read consonant clusters such as /flo/ or mixed open, closed syllables such as /dor/ in words like /flotador/, and reverse syllables such as /al/ or /en/ in utterances such as /al recién nacido/. Almost none of the children in the classroom are able to write a word themselves. Thus, copying words or phrases is the most common task that children carry out in class.

Personal relationships among children are almost nonexistent in the classroom. Children do not have friends in class (except for their relatives). They do not appear as empathic or affectionate towards other children. Children themselves penalize positive behaviors because they detract their dominant position in front of other children. Physical contact among children is scarce, and aggressions or disputes over occupying a physical space or using a given school supply are common. Gender differences are clearly marked and are accepted by everybody. In the same way, students exhibit premature behaviors of adolescence or their coming of age as boys or girls. The male and female students are both mindful of their appearances (particularly their hairstyle) and use highly sexual gestures and words. Literacy in this adverse context is exceptionally complicated. The involvement of teachers with their respective work is commendable. They try to find activities that favor children’s literacy, repeatedly seek the participation of children, and try to involve families and the community by approaching the roman culture.

In relation to the agents involved in ethnographic research, two of the authors of the article acted as observing participants in the study. In this role, we helped the teachers in the classroom, marking student activities, following the teachers’ instructions to help guide the students while they worked in smaller groups, pointing out reading errors, watching over them during recess or accompanying them during trips away from the school.

### Data collection

This study was reviewed and approved by the Ethical Committee of Experimentation (EC). Following the recommendations of the ethics committee, the following measures were taken:

1. All names of participants were changed and the name of the school was omitted to make the study anonymous.
2. The photographs were pixelated so that the study participants would not be recognized. 3. Consent was obtained from the parents and guardians of the minors included in the study and from the school principal.

It took two years to complete the field work. Of these two years, a term was devoted to track the radio program activities. Data collection in different spaces (classroom, hallway, radio cabin, playground) was carried out by following the mosaic approach [29, 30]. Thus, the written productions of students, drawings, radio scripts, pictograms, photographs and audio and video recordings were collected. Likewise, interviews were conducted with the teacher in charge of radio, the classroom teacher, and the students. See Table 1 to know about the quantity and length of these records.

**Table 1:** Data collection procedures.

Data collection	Number	Time
Reports	15	
Audio recordings	25	4h 52’ 04”
Video recordings	39	3h 11’ 45”
Photographs	912	

Data analysis was carried out within the framework of social semiotics [31] to ensure that a social semiotic multimodal approach was adopted while analyzing the social process of meaning-making. In that approach, the focus is on the signs made in response to a prompt in a specific social environment [18]. With regard to the environment of the radio program, we analyze how the signs incorporate the features of the prompt, as well as the resources brought by the makers of the new signs (teachers, children), and how each sing-maker assesses the environment (for instance, ‘in a cabin radio’), in which the sign is to act as a message [32]. The investigators acted as observing participants and were in contact with the children and teachers for a long period of time (twice a week for two school years), which allowed the children to behave naturally and interact with their peers and teachers without the awkwardness of being observed by strangers. This prolonged engagement, along with the persistent observation in the field, triangulation of different methods (observations, interviews, student assignments, documents, etc.), researchers and data contribute to an increased likelihood of credible results. Likewise, the interpretation of the information collected was carried out through a triangulation of sources: classroom observation, analysis of videos, photographs, audios and materials prepared by the students. The interviews with both teachers and students allowed us to perform a members check, which favors the communicative validation of data and the participants’ interpretations [25]. The conclusions reached by the investigators after their observation of the literacy event were contrasted with the opinions of the participants in aspects such as: the choice of the subject matter of the radio program, the nature of the children’s participation in the making of the script, the children’s ability to memorize said script when they were not able to read it, the management of the program’s emission by the teacher, the lack of interest that the children displayed for the radio program, or the help provided by the teacher when it was time for the kids to complete the related activities, after the program had been aired.

Amongst the different methods of information harvesting mentioned, interviews and observations are the most useful, as it is possible to access their content through the transcriptions of the audios and videos taken during class and extracurricular activities. A good move during the data harvesting process was the decision to switch from video cameras and recorders to mobile phones, which generated no rejection in the children or teachers, as it is technology they are familiar with. In particularly vulnerable contexts, such as the ones in our study, the use of mobile phones favors uninhibition, even though the participants were aware they were being filmed. On the other hand, it was crucial to include all the multimodal signs that reflect the affection and dislike generated by the exchanges at school when making the transcriptions, in order to develop the semiotic analysis that this investigation defends.

## Results and discussion

The open radio broadcasts take place periodically at the school itself; although, their frequency does increase progressively based on students’ age. While the eleven- and twelve-year-old students take part in radio programming and production and listening activities on a weekly basis, the younger students take part in only one broadcast throughout the entire school year. A radio broadcast is conducted in line with a typical sequence that entails selection of the radio program theme, student involvement as “editors” and “broadcasters”, the preparation of a program script, rehearsal, and the broadcast of the program. At the beginning of the year, the teaching staff determines each of the program themes and the dates when these programs would be broadcasted for each group of students. Neither the students nor the families take part in this process. The programs are broadcasted in every classroom live through a loudspeaker service. These broadcasts may also be listened to at a later time, say during the afternoon and outside the school, as they are available to the entire neighborhood through FM dial.

The following paragraphs deal with the most controversial aspects, illustrating how the literacy event fails to attain an affective encounter in two of its most relevant moments. Besides, the elements that contribute toward power inequality, vis-à-vis the literacy process, and the failure to resort to the cultural capital of the event’s participants have been emphasized.

### Brainstorm analysis: Nondialectical assembly

In the case under study, the script for the radio program is written by the class teacher based on the topic previously agreed by the teaching staff. The teacher presents the topic to the class: “Who knows what we are scheduled to do for the radio program?” and tries to introduce a brainstorming activity to elicit responses from the students. The proposed topic, “Book Week,” is totally unknown to the students, who end up asking the teacher, “What is Book Week?” While one child associated “Book Week” with short story listings, another child associated it with storyteller (both of which are activities that the school organizes every year). The rest of the students did not get involved.

The brainstorming session takes place in the framework of an assembly. The children and the teacher sit together at the same height using three long benches in a truncated pyramid layout. They are sitting in proximity and in a position that allows each one of them to see the faces of each other, and move, stand up, and often change posture. At the level of discourse, as depicted in Table 2, 9 out of 17 of the teacher’s oral interventions, are devoted toward imparting instructions/directions, above all in relation to the behavior expected of students to take part in the radio program.

**Table 2:** The teacher’s and students’ communicative shifts in classroom assembly regarding the radio program theme.

Summary of the meanings made in different modes			
Time	Speech	Action	Visual
9.00.03	(T)’s question to be answered by students on the agenda	(S) remain calm	Lecturer’s notebook
9.00.11	(S)’ answer on the agenda	(s) makes a fake surprise	
9.00.18	(T) presents the radio program instructions	(T) controls	
9.00.20.	(s) asks for participation in the radio program	(s) joins hands, as in a prayer	
9.01.03	(T) presents the rules to take part in the radio program–warning, persuasion	(T) physically controls the students next to her (she grabs arms and turns heads)–keeping attention toward her discourse	
	(s) assents	(S) gesture tiredness, yawning	
9.01.11	(T) presents the rules for the process of preparing the radio program theme	(T) orders students to be silent (she hushes them)	
9.01.17	(T) persuades on the importance of following rules for the topic to be properly prepared. Example of similar situations–hard work	(S) gesture tiredness, yawning–boredom	
9.01.27	(s) Repetition of the hard work idea	(s) formal posture–importance of the idea	
9.01.32	(T) poses a rhetorical question concerning the radio program topic		
	(S) give random answers	(S) raise hands–request for intervention	
9.01.37	(T) Informs of the program topic [Book Day]	(S) gesture tiredness, yawning–boredom	
		–No reaction to the announcement	
9.01.43	(s) asks for information on the topic	(T) marks the name of the girl who asks	Lecturer’s notebook
9.01.48	(T)’s instructions–search for information about the program topic	(T) is taking notes	Lecturer’s notebook
9.01.50	(s) provides input in connection to the topic	(s)’s formal posture on the importance of the idea	
		(S) gesture boredom	
9.02.09	(T) questions on the importance of books	(s) stands up from the desk	
	(s) answers on the importance of books	(S) whisper tiredness	
9.02.40	(T) rule–take turns	(T) on hand raising–request for intervention, physical control of students next to her	
		(S) on hand raising–request for intervention	
9.02.55	(s) provides input on the importance of books	(T) takes notes	Lecturer’s notebook
	(T) agrees to student’s input	(s) keeps hand raised	
9.03.07	(s)’s demand to the lecturer unrelated to the radio program	(s) stands up–asking for a fabric bracelet to be cut	
		(T) cuts the fabric ribbon using scissors	
9.03.09	(T) gives the floor–asking students about the topic	(s) lowers the hand	
		(S) gesture boredom	
9.03.32	(s)’s general input on the text read	(T) looks at student–attention	
9.03.48	(s) complaints of sore throat	(s) weeps	
	(T) listens–degrades the importance of pain	(S) stand up, gesture of tiredness	
9.03.56	(T) reproaches the student for her recurring behavior–calling her mother to go home	(T) shows her the watch–reinforcing the idea that it is early to call the student’s mother to come and pick her up	
9.04.23	(s) ask permission to use the toilet	(S) stand up and move	
	(s) question outside the program theme	(T) disregards irrelevant questions–addressing her words toward and fixing her eyes on the student who claims to be sick and asks to use the toilet	
	(T) recalls the rule to use the toilet–reproaching the student for her behavior		
9.04.27	(T) gives the student the permission to use the toilet–justifying her decision	(s) Leaving the classroom	
		(S) stand up and move	
9.04.45	(T) redirects the situation and discusses the program’s theme	(S) stand up and move	Teacher’s notebook
	(s) suggests concerning the topic	(T) note-taking	
	(S) answering their classmates–talking all at once		
9.05.15	(T) rule–turns to take part	(T) gestures to emphasize the message	

(T) teacher; (s) a student; (S) students.

The right to go to the radio is granted by the teacher based on her values: 0.24–0.41 (T) “It is mainly children who can read, who will be able to attend the radio program and read. Above all, it is those who earn the right to go, those who make an effort and show good manners. Mind you: a person who reads super nicely, but misbehaves, will not go to the radio program, that is for sure.” 0.53–0.58 (T). The symbolic capital [33] allocated to behavior rules and reading abilities on the basis of their emergence is contradictory to the reality of children whose families have a different kind of literacy, belonging to a “peripheral normativity,” [34] and, thus, possess a very low orthodox level of writing and reading skills (only one of all of the children’s mothers had completed her primary education). As a result, they have unequal access to the main literacy event [35] during the assembly. Besides, if the relationship between the ideational and the interpersonal levels [21] of Book Day consideration is analyzed, children’s responses to the teacher’s question: “Do you believe books to be important in school?” are meaningful: 7.16- (s). We could read without books (the student repeats this three times); 7.19- (s). They are not [important] because I write something on a piece of paper and now we can read it. There you go; 8.36- (s). If we do not have any books, the teacher could write on the board and we could read.

Additionally, the teacher’s arguments concerning the need for reading books refer to the mechanical aspects of the instance of reading: 6.40–6.50 (T) “There are lots of things we can use to learn to read, but books are important to help us practice, gain speed, and improve our reading skills.” Or it may refer to a merely referential function: 2.42 (T) “They are important to obtain information about things.” At no time does the teacher allude to the affective quality of literacy [16] or to its relationship with pleasure [32]. No textbooks are used in the classroom either. The books present in the classroom are classical short stories that have not been adapted in accordance with the children’s reading skills. Following the same line of thought, at the exclusively textual level, the scripts written for the radio do not convey the identity of the neighborhood’s culture [32]. Thus, if we attempt to approximate the “ideal” level, as stipulated by the discourse, to the “real level” inherent in the classroom’s idiosyncrasies, we find total inconsistency.

Moreover, the students in the assembly do not exhibit active listening skills, as only three of the teacher’s 17 interventions involved posing questions to the students. What is more, one such intervention is clearly rhetoric and not susceptible to being answered by the students. The rest of her interventions are informative and/or persuasive. The assembly adopts a monologic structure, in opposition to the concept of assembly, as it appears to follow a script: classroom discourse is monologic to the extent that the teacher is usually guided by a predetermined script [33].

At the multimodal level, the teacher solely relies on her notebook, in which she apparently takes notes of students’ contributions. Such notetaking is basically a pretense: it is found that none of the students’ suggestions are taken into account for the radio script. At the action level, children’s lack of interest in and boredom with the chosen theme is visible with their yawning, tiredness gestures, and repeated shuffling around the classroom, disregarding the teacher’s discourse (Table 2). It is worth noting in this respect that at no time did the teacher decide to change the tactics in the assembly by adopting another type of literacy resource, which would be apt for students’ day-to-day experience. The teacher turned her back toward any allusion of existing literacy that lied outside the school [34].

In terms of power distribution, at the end of the brainstorming session, the selection of the four children who will take part in the radio program is completed based on the criteria established by the teacher, considering 0.53–0.58 (T): “Who is going there? Remember that it is a prize, it is an honor to take part in the radio program.” This turns the “chosen” ones into legitimate broadcasters and agents, privileged by the power status granted by normative reading and writing literacy, whilst the rest of the children are rendered invisible. Given their social exclusion, this provides them with power categories based on cultural capital [33], which is available to them only within the context of the school. This being said, these children also become anonymous beings the moment they lose these behavioral and reading and writing skills, with the subsequent fragility of their sense of belongingness to the educational system. The relational dimension inherent in literacy is totally lost [35]; the reading and writing instance thus, becomes a competitive goal that solely belongs to the academic field.

### Analysis of the program broadcast: Command control

The day when the radio program is recorded, the children who take part as broadcasters arrive earlier than usual. The radio teacher comes to the classroom ahead of the starting time and interacts with the children. Meanwhile, the tutor explains to her that they arrive late every day, however, have been very punctual today. The tutor especially congratulates one of the children who is always extremely late, “Even my JC who always arrives at 10:00 am has been early today.” (0.19) They all engage in talking to each other, whilst the tutor finishes cutting and sticking the pieces of the script on a piece of cardboard.

**Fig 1:**
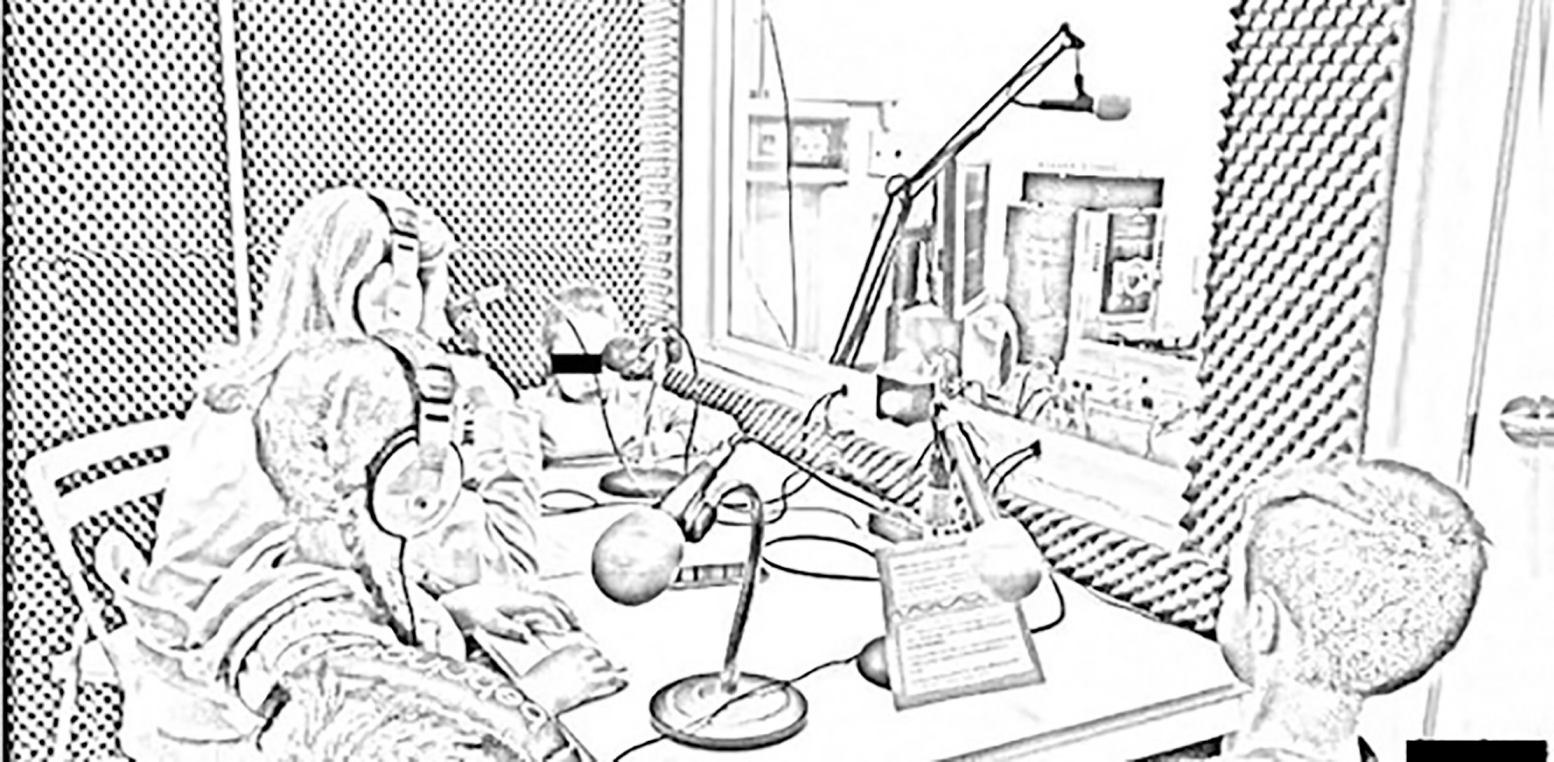
Photograph of the radio booth.

The broadcasters leave towards the studio, while the rest of the children stay in the classroom with another teacher who will step in for the tutor during the recording of the program. The teachers use this time to impart last instructions and answer their questions while they wait. Lots of comments arise, the children’s interests are piqued by the technical aspects surrounding the recording. The children continue to express nervousness and are disquiet (they jump, shuffle, some go to the toilet, all of them, except J., talk a lot). Each one of them is still sitting on a chair outside the booth; they wait, whilst the teacher talks to the operator who will operate the recording dashboard. During this time, the children are waiting silently. They finally get into the radio booth, sit down, and the radio teacher imparts instructions on how to place the headbands, the microphone, and the script (Fig 1).

An initial discourse analysis reveals that the radio teacher had 26 interventions, while the children had only 11 interventions (Table 3).

**Table 3:** The teacher’s and students’ communicative shifts between modes in the radio program broadcasting.

Summary of the meanings made in different modes			
Time	Speech	Action	Visual
9.25.00	(rT) imparts detailed and repetitive instructions to children concerning how and where to sit	(rT) points her finger, grabs children from their shoulders, places them in their positions, and pushes their chairs until they are close to the table	
9.26.17	(rT) persuades children to sit properly by narrating the importance of sitting properly	(S) each child sits in their chair by following the teacher’s instructions	
9.26.55	(rT) prescribes how chairs must be moved and repeats it several times	(rT) by doing it herself first, shows children as to how they must move their chairs to take them closer to the table, and moves some children’s chairs and pushes the children closer to the table	
		(S) move their chairs by mimicking the teacher’s movements	
9.26.57	(s) says that she has done as instructed by the teacher	(rT) pushes the children’s chairs forward until they are close to the table	
	(rT) gives the girl positive feedback		
9.27.22	(rT) prescribes on how to sit and how not to sit on chairs	(rT) checks that all the chairs are properly positioned and moves those that still are not; she further checks that children are sitting correctly using her body to show improper postures that should not be maintained	
9.27.26	(T) imparts instructions to a child as to where to place the script on the table to read it easily	(T) grabs a child’s script and positions it on the table close to the child and the microphone	Program script for each child
9.27.33	(rT) instructs children on how to wear headphones and asks them if they intend to wear headphones	(rT) holds up the headphones	
		(S) mimic her actions and nod in response to the teacher’s question	
9.28.06	(rT) establishes that a set of headphones must be shared between the two girls and asks them to organize in a manner where they can take turns	(S) do as the teacher instructs	
	(S) suggests a shared use of headphones		
9.28.09	(rT) reproaches one of the children for having already put on the headphones	(rT) turns her body toward the children	
	(T) admonishes the child	(s) takes off the headphones	
9.28.36	(rT) repeats with the other three children that they must share two sets of headphones and asks them as to how they are going to share them	(T) makes hand gestures and approaches the table to attract the girls’ attention	
		(rT) turns in the direction of the two girls to reinforce the idea that it is the girls who must decide	
	(S) explain how they plan to share the headphones		
	(T) admonishes the two girls who are talking to each other		
	(rT) rebuts		
	(T) defends the need for the girls to talk to each other and decide how they will share their headphones		
9.29.12	(rT) argues that the three children must share the two sets of headphones, asks a rhetorical question about the children’s desire to wear the headphones, and asks them to explain how they intend to share the headphones	(rT) makes hand gestures to reinforce her arguments and turns to face the children to whom she is talking to	
		(S) don’t move or gesticulate–they appear submissive to the teacher’s words	
	(S) suggests on how they should share the headphones and (rT) repeatedly objects against the students’ suggestions		
	(T) supports (rT)’s arguments		
9.30.53	(rT) explains the manner in which she considers that the children should share the headphones and asks them on how they would do it (S) the children appear to be in agreement	(rT) looks at each of the children to check if they are following her explanations and makes hand gestures to reinforce her message	Radio program script
		(o) follows (rT)’s instructions	
		(s) gestures of tiredness	
		(S) nod their heads in assent	
9.31.37	(rT) asks the children as to the order in which they will use the shared headphones	(S) put on the headphones	
		(rT) distributes headphones to children and helps them tighten them	
	(S) state the order in which they will share the headphones		
9.31.53	(rT) asks children to take off the headphones and listen to her	(S) take off the headphones again	
	(rT) instructs on how to handle the microphones and the distance that they are required to keep	(T) snatches the headphone set from one of the children’s hands and puts them on the table–whispering an admonishment	
9.32.40	(rT) imparts instructions as to how the recording will start and the order of interventions	(rT) changes her tone of voice to underscore the most important instructions	
		(rT) makes gestures, trying to emphasize what the children must not do once the recording starts	
	(S) repeat the teacher’s instructions		
	(T) repeats (rT)’s idea		
9.33.25	(rT) imparts instructions as to how to talk into a microphone	(rT) handles the microphones and puts on the headphones	
	(T) tells the children to read in a loud and clear voice, for them to be heard	(S) put on the headphones	
		(rT) starts the timer in her cell phone and checks to see if all children are properly situated and ready to start	
9.33.59	(rT) starts reading the script	(s) moves her head to greet	
	(rT) greets the operator		
	(o) greets back		
	(rT) asks a girl to say good morning to the audience, reproaches her for doing so with a gesture rather than doing it orally, and argues that the radio is for speaking		
	(s) repeats the greeting orally and the (rT) congratulates her for that		
9.34.23	(rT) greets the children one by one and asks them to greet back	(S) approach the microphone and speak	
	(S) greet		
9.34.24	(rT) persuades the audience that one of the children is deeply interested in the program and asks the student	(rT) brings the microphone closer to her	
		(s) approaches the microphone	
		(T) makes a greeting gesture	
	(s) answers affirmatively		
	(rT) greets (T) and persuades the audience that the children are behaving well		
	(T) says good morning		
9.37.47	(rT) presents the first girl’s intervention	(s) looks at the teacher before starting to read–she reads syllable by syllable	Program script
	(s) asks the teacher for permission and reads the script		
9.38.24	(s) reads her part of the script	(rT) and (T) position the script at the level of the eyes of the child who is reading	Program script
		(rT) positions the script at the level of the eyes of the following child who is going to read–the child reads syllable by syllable	
9.39.14	(s) reads the script	(rT) and (T) help children position the script, so that it is easier for them to read it	Program script
		(s) reads syllable by syllable, uses a finger as a guide to read	
9.40.41	(s) reads the script	(rT) nods her head to encourage the child who is reading-goes syllable by syllable	Program script
		(rT) gesticulates to the (o), asking him to pause the recording	

(rT) radio teacher; (T) teacher; (s) a student; (S) students; (o) radio operator.

At any rate, if we analyze the quality of these interventions, we can examine how most of the time, the radio teacher uses a prescriptive speech (11/26), persuasive speech (7/26), and reproachful speech (2/26) with the support of the main tutor, who is participating only to control the children’s behavior. She also asks several “quasi-authentic” questions [36], which are similar to examination questions, in so far as they entail an asymmetrical relation in which the teacher expects a given answer. Meanwhile, the students devoted their speeches to repeating instructions (4/11), asking permission (2/11), reading the script written by the teacher (3/11), giving their assent (2/11), and suggesting and arguing in favor of their turn with the headphones (3/11). However, this latter event shall be a linguistic exercise, rather than a dialogic one, in as much as the radio teacher will be making decisions in that respect (9:31:53), along with an univocal answer that annuls the flow of questioning. This means that at no time are the students given the chance to use their speech creatively [37]. They are constrained to utter what must be said and made to abstain from what must not be said [38] via indirect control.

At the body language level, the control over children is even more clear. The teacher’s interventions are mostly geared towards ensuring that her rules are obeyed, going as far as moving her body and the furniture against the side that the children are leaning towards to lead the action. The students, in turn, appear to be submissive and attentive to the instructions. Given the concept of performative action described by McLaren [39] inside educational establishments, this case illustrates how this “radio ritual” is imposed by the teacher on children in a ritualistic enactment of bodies’ modes of being [40]. This is accompanied by a reproduction dynamic that ensures dominance in the educational process: “Once this indicator turns orange, nobody moves, nobody talks and nobody does anything, we have the chair already positioned and we do not move at all. Are you ready? (S) Yes (rT), Are you sure? (S) Yes (rT), Are you 100% sure?” (09:20).

The results obtained show that the children are taught reading and writing skills in the framework of a dominant model that does not take into consideration the knowledge funds that arise within the families and in the community. The establishment ends up defining what, how, and why reading and writing skills must be acquired, and exerts its power through the decisions made in connection with the literacy event. A literacy event, even when provided in good faith, can become an alternative mode through which school exercises its power and imposes its literacy model on a community. This dominant model does not train students or their families when their starting point is a culture that makes the students and their families perceive the model as alien and something that is forced upon them.

## Conclusions

A look at all these aspects in connection with power allows us to assert that, at the interpersonal level, social roles are well established: dominant and dominated, with the latter being supported at an ideational level for configuration of the event’s instructions; and at the textual level with a basically referential script. There is a coupling of artificial, unnatural structures built for the benefit of a mode of being, of thinking, and of a particular group, in this case, made up by the teaching staff. It is a social construct of an upper-middle class that privileges and values an understanding of the world, which is stipulated as universal. Thus, the mediated action [36] in the educational process takes place from a given place that is alien to the recipients of the said process.

Despite the establishment’s and the teacher’s good intentions, the approach to literacy teaching and its methods is not appropriate and has poor results. In fact, literacy is understood as an event that does not occur, while no “affective” space of interaction is created between the children and adults. Neither is it created with the text. These three premises are suggested for generating a literacy event [31, p45]:

1. Event is generated as people and things come into relation
2. What happens always exceeds that which is conceived and perceived
3. Implicit in the event are multiple potentialities, including multiple possibilities for what might materialise as well as what does not

All the elements are controlled in this experience, without any space for any potentiality that exceeds the possibilities of the event. No actual encounter occurred among participants. Neither is there any reinforced bond to take advantage of the dialogic space offered by the radio. It is in opposition to the affective perspective of Leander and Ehret [16] which proposes that literacy teaching should be used “to create, sustain and extend events and relationalities that are enfolded into the collective” [p225], more so in environments that are at risk of social exclusion. To create anti-hegemonic practices, opportunities should be given to actively hook students: objects, tools, and beneficiaries’ own knowledge [16]. The practices analyzed in this paper are against a type of literacy that is urgently calling for sustenance. Their rules and standards turn out to be more fluid and perhaps more ephemeral than those associated with established literacies [11].

Due to the Covid-19 pandemic, it has not been possible to arrange a meeting with both the parents and the center, since there are movement restrictions in place for those who do not belong to the community. However, we have arrived at the following conclusions, which will be explained in due course. In terms of bettering the education, it is notable how this investigation’s analysis questions the lack of coherence of a completely outdated pedagogy of literacy. If we were to dig deeper, we might even point out the lack of attention paid to the immateriality of the education processes, and, therefore, the lack of awareness over how students react to strategies that they do not find meaningful. This corroborates that current education strategies are not really focused on the student body. Educational praxis must thus be focused on less controlled environments, which will allow literacies to exist from spatial, corporeal and even digitally mediated perspectives [41]. Education agents (school boards, faculty, families) must rethink literacies in the context of community, transforming literacy into a collaborative effort [42]. An example of these new practices would be to make use of repositories that take into account funds of knowledge [43], popular culture, and the need to avoid imposing subject matters. It is also essential to create a bridge between families and schools, introducing that third space into the methodologies and contents for a more open curriculum. This pushes the realities that are used as reference in the classroom beyond merely representational concepts. We would achieve an understanding of education with a post-human emphasis, related to knowing, becoming, and doing literacies [44].

## Supporting information

S1 Table Data collection procedures. (TIF)

S2 Table The teacher’s and students’ communicative shifts in classroom assembly regarding the radio program theme. (PDF)

S3 Table The teacher’s and students’ communicative shifts between modes in the radio program broadcasting. (PDF)
