# Supplementary figures and images for: Literacy, power, and affective (dis)encounter: An ethnographic study on a low-income community in Spain
Source: PLoS One. 2021 Jun 4;16(6):e0252782. doi: 10.1371/journal.pone.0252782 (PMC8177657; doi:10.1371/journal.pone.0252782)

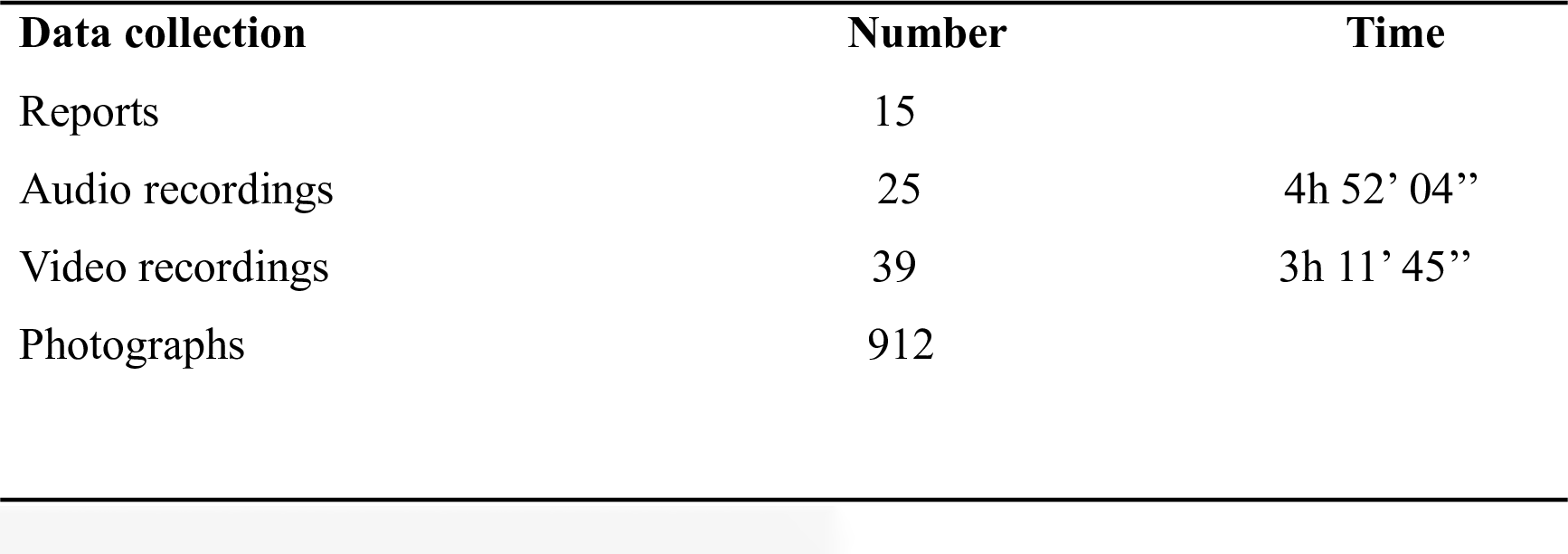

Supplement: S1 Table — (TIF) [file pone.0252782.s001.tif]
