# Supplementary material for: Literacy, power, and affective (dis)encounter: An ethnographic study on a low-income community in Spain
Source: PLoS One. 2021 Jun 4;16(6):e0252782. doi: 10.1371/journal.pone.0252782 (PMC8177657; doi:10.1371/journal.pone.0252782)
Supplement: S2 Table — (PDF) [file pone.0252782.s002.pdf]

Summary of the meanings made in different modes			
Time	Speech	Action	Visual
9.00.03	(T)'s question to be answered by students on the agenda	(S) remain calm	Lecturer's notebook
9.00.11	(S)' answer on the agenda	(s) makes a fake surprise	
9.00.18	(T) presents the radio program instructions	(T) controls	
9.00.20.	(s) asks for participation in the radio program	(s) joins hands, as in a prayer	
9.01.03	(T) presents the rules to take part in the radio program–warning, persuasion<br><br>(s) assents	(T) physically controls the students next to her (she grabs arms and turns heads)–keeping attention toward her discourse<br>(S) gesture tiredness, yawning	
9.01.11	(T) presents the rules for the process of preparing the radio program theme	(T) orders students to be silent (she hushes them)	
9.01.17	(T) persuades on the importance of following rules for the topic to be properly prepared. Example of similar situations–hard work	(S) gesture tiredness, yawning–boredom	
9.01.27	(s) Repetition of the hard work idea	(s) formal posture–importance of the idea	
9.01.32	(T) poses a rhetorical question concerning the radio program topic<br>(S) give random answers	(S) raise hands–request for intervention	
9.01.37	(T) Informs of the program topic [Book Day]	(S) gesture tiredness, yawning–boredom –No reaction to the announcement	
9.01.43	(s) asks for information on the topic	(T) marks the name of the girl who asks	Lecturer's notebook
9.01.48	(T)'s instructions–search for information about the program topic	(T) is taking notes	Lecturer's notebook
9.01.50	(s) provides input in connection to the topic	(s)'s formal posture on the importance of the idea<br>(S) gesture boredom	
9.02.09	(T) questions on the importance of books<br>(s) answers on the importance of books	(s) stands up from the desk<br>(S) whisper tiredness	
9.02.40	(T) rule–take turns	(T) on hand raising– request for intervention, physical control of students next to her<br>(S) on hand raising– request for intervention	
9.02.55	(s) provides input on the importance of books<br>(T) agrees to student's input	(T) takes notes<br>(s) keeps hand raised	Lecturer's notebook
9.03.07	(s)'s demand to the lecturer unrelated to the radio program	(s) stands up–asking for a fabric bracelet to be cut<br>(T) cuts the fabric ribbon using scissors	
9.03.09	(T) gives the floor–asking students about the topic	(s) lowers the hand<br>(S) gesture boredom	
9.03.32	(s)'s general input on the text read	(T) looks at student–attention	
9.03.48	(s) complaints of sore throat<br>(T) listens–degrades the importance of pain	(s) weeps<br>(S) stand up, gesture of tiredness	

9.03.56	(T) reproaches the student for her recurring behavior–calling her mother to go home	(T) shows her the watch–reinforcing the idea that it is early to call the student's mother to come and pick her up	
9.04.23	(s) ask permission to use the toilet<br>(s) question outside the program theme<br>(T) recalls the rule to use the toilet–reproaching the student for her behavior	(S) stand up and move<br>(T) disregards irrelevant questions–addressing her words toward and fixing her eyes on the student who claims to be sick and asks to use the toilet	
9.04.27	(T) gives the student the permission to use the toilet–justifying her decision	(s) Leaving the classroom<br>(S) stand up and move	
9.04.45	(T) redirects the situation and discusses the program's theme<br>(s) suggests concerning the topic<br>(S) answering their classmates–talking all at once	(S) stand up and move<br>(T) note-taking	Teacher's notebook
9.05.15	(T) rule–turns to take part	(T) gestures to emphasize the message	

(T) teacher; (s) a student; (S) students
