# Supplementary material for: Literacy, power, and affective (dis)encounter: An ethnographic study on a low-income community in Spain
Source: PLoS One. 2021 Jun 4;16(6):e0252782. doi: 10.1371/journal.pone.0252782 (PMC8177657; doi:10.1371/journal.pone.0252782)
Supplement: S3 Table — (PDF) [file pone.0252782.s003.pdf]

| Summary of the meanings made in different modes | | | |
|---|---|---|---|
| Time | Speech | Action | Visual |
| 9.25.00 | (rT) imparts detailed and repetitive instructions to children concerning how and where to sit | (rT) points her finger, grabs children from their shoulders, places them in their positions, and pushes their chairs until they are close to the table | |
| 9.26.17 | (rT) persuades children to sit properly by narrating the importance of sitting properly | (S) each child sits in their chair by following the teacher's instructions | |
| 9.26.55 | (rT) prescribes how chairs must be moved and repeats it several times | (rT) by doing it herself first, shows children as to how they must move their chairs to take them closer to the table, and moves some children's chairs and pushes the children closer to the table<br>(S) move their chairs by mimicking the teacher's movements | |
| 9.26.57 | (s) says that she has done as instructed by the teacher<br>(rT) gives the girl positive feedback | (rT) pushes the children's chairs forward until they are close to the table | |
| 9.27.22 | (rT) prescribes on how to sit and how not to sit on chairs | (rT) checks that all the chairs are properly positioned and moves those that still are not; she further checks that children are sitting correctly using her body to show improper postures that should not be maintained | |
| 9.27.26 | (T) imparts instructions to a child as to where to place the script on the table to read it easily | (T) grabs a child's script and positions it on the table close to the child and the microphone | Program script for each child |
| 9.27.33 | (rT) instructs children on how to wear headphones and asks them if they intend to wear headphones | (rT) holds up the headphones<br>(S) mimic her actions and nod in response to the teacher's question | |
| 9.28.06 | (rT) establishes that a set of headphones must be shared between the two girls and asks them to organize in a manner where they can take turns<br>(S) suggests a shared use of headphones | (S) do as the teacher instructs | |
| 9.28.09 | (rT) reproaches one of the children for having already put on the headphones<br>(T) admonishes the child | (rT) turns her body toward the children<br>(s) takes off the headphones | |
| 9.28.36 | (rT) repeats with the other three children that they must share two sets of headphones and asks them as to how they are going to share them<br>(S) explain how they plan to share the headphones<br>(T) admonishes the two girls who are talking to each other<br>(rT) rebuts<br>(T) defends the need for the girls to talk to each other and decide how they will share their headphones | (T) makes hand gestures and approaches the table to attract the girls' attention<br>(rT) turns in the direction of the two girls to reinforce the idea that it is the girls who must decide | |
| 9.29.12 | (rT) argues that the three children must share the two sets of headphones, asks a rhetorical | (rT) makes hand gestures to reinforce her arguments and turns to face the children to whom she is talking to | |

| | | | |
|---|---|---|---|
| | question about the children's desire to wear the headphones, and asks them to explain how they intend to share the headphones<br>(S) suggests on how they should share the headphones and (rT) repeatedly objects against the students' suggestions<br>(T) supports (rT)'s arguments | (S) don't move or gesticulate–they appear submissive to the teacher's words | |
| 9.30.53 | (rT) explains the manner in which she considers that the children should share the headphones and asks them on how they would do it<br>(S) the children appear to be in agreement | (rT) looks at each of the children to check if they are following her explanations and makes hand gestures to reinforce her message<br>(o) follows (rT)'s instructions<br>(s) gestures of tiredness<br>(S) nod their heads in assent | Radio program script |
| 9.31.37 | (rT) asks the children as to the order in which they will use the shared headphones<br>(S) state the order in which they will share the headphones | (S) put on the headphones<br>(rT) distributes headphones to children and helps them tighten them | |
| 9.31.53 | (rT) asks children to take off the headphones and listen to her<br>(rT) instructs on how to handle the microphones and the distance that they are required to keep | (S) take off the headphones again<br>(T) snatches the headphone set from one of the children's hands and puts them on the table–whispering an admonishment | |
| 9.32.40 | (rT) imparts instructions as to how the recording will start and the order of interventions<br>(S) repeat the teacher's instructions<br>(T) repeats (rT)'s idea | (rT) changes her tone of voice to underscore the most important instructions<br>(rT) makes gestures, trying to emphasize what the children must not do once the recording starts | |
| 9.33.25 | (rT) imparts instructions as to how to talk into a microphone<br>(T) tells the children to read in a loud and clear voice, for them to be heard | (rT) handles the microphones and puts on the headphones<br>(S) put on the headphones<br>(rT) starts the timer in her cell phone and checks to see if all children are properly situated and ready to start | |
| 9.33.59 | (rT) starts reading the script<br>(rT) greets the operator<br>(o) greets back<br>(rT) asks a girl to say good morning to the audience, reproaches her for doing so with a gesture rather than doing it orally, and argues that the radio is for speaking<br>(s) repeats the greeting orally and the (rT) congratulates her for that | (s) moves her head to greet | |
| 9.34.23 | (rT) greets the children one by one and asks them to greet back<br>(S) greet | (S) approach the microphone and speak | |
| 9.34.24 | (rT) persuades the audience that one of the children is deeply interested in the program and asks the student<br>(s) answers affirmatively<br>(rT) greets (T) and persuades the audience that the children are behaving well<br>(T) says good morning | (rT) brings the microphone closer to her<br>(s) approaches the microphone<br>(T) makes a greeting gesture | |

| | | | |
|---|---|---|---|
| 9.37.47 | (rT) presents the first girl's intervention<br>(s) asks the teacher for permission and reads the script | (s) looks at the teacher before starting to read–she reads syllable by syllable | Program script |
| 9.38.24 | (s) reads her part of the script | (rT) and (T) position the script at the level of the eyes of the child who is reading<br>(rT) positions the script at the level of the eyes of the following child who is going to read–the child reads syllable by syllable | Program script |
| 9.39.14 | (s) reads the script | (rT) and (T) help children position the script, so that it is easier for them to read it<br>(s) reads syllable by syllable, uses a finger as a guide to read | Program script |
| 9.40.41 | (s) reads the script | (rT) nods her head to encourage the child who is reading-goes syllable by syllable<br>(rT) gesticulates to the (o), asking him to pause the recording | Program script |

(rT) radio teacher; (T) teacher; (s) a student; (S) students; (o) radio operator
